# ARumenamides as Multitarget Ion Channel Modulators: Insights from Fenestration-Focused Docking, ADMET Profiling, and Molecular Dynamics

**DOI:** 10.3390/ijms27114786

**Published:** 2026-05-26

**Authors:** Mena Abdelsayed, Yassir Boulaamane

**Affiliations:** 1 Lankenau Institute for Medical Research, Philadelphia, PA 19096, USA; 2 Laboratory of Innovative Technologies, National School of Applied Sciences of Tangier, Abdelmalek Essaadi University, Tetouan 93000, Morocco; boulaamane.yassir@etu.uae.ac.ma

**Keywords:** voltage-gated ion channels, polypharmacology, molecular dynamics simulations, ARumenamides

## Abstract

Voltage-gated ion channels are central regulators of cardiac, neuronal, and skeletal muscle excitability, and their dysfunction underlies a wide spectrum of channelopathies, including arrhythmias and neuromuscular disorders. While conventional ion channel therapeutics typically target a single pore-binding site, emerging evidence supports the therapeutic potential of polypharmacological compounds capable of modulating multiple channel subtypes. ARumenamides represent a novel class of sulfonamide-based ligands originally identified as fenestration-targeting sodium channel modulators; however, their cross-family binding mechanisms and multitarget potential remain incompletely defined. Here, we employed an integrated structure-based computational workflow combining molecular docking, in silico ADMET profiling, and long-timescale (250 ns) molecular dynamics simulations to systematically evaluate 20 ARumenamide derivatives across 15 voltage-gated sodium, calcium, and potassium channel structures. Docking analyses revealed broad multitarget binding profiles, with several compounds exhibiting high predicted affinity across cardiac, neuronal, and skeletal muscle channel isoforms. ADMET predictions demonstrated favorable intestinal absorption and metabolic safety for most candidates, although solubility and mutagenicity liabilities were identified for select derivatives. Detailed molecular dynamics simulations of prioritized compounds (AR-310, AR-769, and AR-946) uncovered site-specific binding behaviors and conformational effects. AR-769 exhibited exceptional stability at both fenestration and central pore sites of Cav1.2, associated with persistent hydrogen-bond networks, reduced protein flexibility, and a well-defined free energy minimum. In contrast, AR-310 and AR-946 displayed selective stability within Nav1.4 fenestrations and the Kv4.3 central pore, respectively, highlighting how subtle chemical features bias binding site preference and dynamic retention. Collectively, these findings establish a structure–dynamics framework for rational design of ARumenamide-based multitarget ion channel modulators. Our results demonstrate that fenestration-focused binding can support sustained ligand engagement without obligatory pore occlusion, offering a mechanistically distinct strategy for developing next-generation polypharmacological therapeutics for cardiac and neuromuscular disorders.

## 1. Introduction

Ion channels are critical regulators of cellular excitability in cardiac, neuronal, and muscle tissues, and dysregulation of these channels underlies a range of diseases from arrhythmias to neuropathic pain [1,2,3]. Cardiac tissues express Nav1.5, Cav1.2, and Kv4.3 (encoded by *SCN5A*, *CACNA1C*, and *KCND3*, respectively), among others [4]. Nav1.4 (*SCN4A*) is the primary skeletal muscle sodium channel, and mutations in *SCN4A* cause periodic paralysis syndromes [5]. Similarly, *CACNA1C* (Cav1.2) mutations lead to multisystem disorders (e.g., Timothy syndrome) involving life-threatening cardiac arrhythmias, and *KCND3* (Kv4.3) gain-of-function variants have been linked to Brugada and early-repolarization syndromes (transient outward current, Ito) [6,7,8]. Together, these channels govern heart rhythm, neuronal firing, and muscle contraction, making them attractive targets for therapeutic modulation [8].

Traditional ion channel drugs typically target a single pore or binding site (e.g., local anesthetics in Nav1.5’s central cavity) [9]. However, modern “polypharmacology” recognizes that multi-target compounds can provide synergistic therapeutic benefits or safety profiles [10,11,12]. For example, a recent study of ARumenamide-787 showed pleiotropic effects: it enhanced Nav1.5 current and inhibited Ito, IKr and Cav currents, suppressing arrhythmias in J-wave syndrome models [13]. Such multitarget ligands may better correct complex channelopathies. In this context, targeting fenestrations, hydrophobic lateral openings in the channel protein, to achieve selective modulation has gained interest [14]. Fenestrations connect the membrane lipid phase to the central cavity and can admit lipophilic drugs even when the gate is closed [15].

Notably, ARumenamides, a novel class of sulfonamide/carboxamide compounds, were originally discovered as sodium-channel modulators that preferentially bind fenestrations. In Nav1.5, bulkier aromatic ARs showed high affinity for III–IV fenestrations, mitigating pore block and loss-of-function phenotypes [16]. However, despite increasing interest in fenestration-mediated binding, a systematic, cross-family evaluation of fenestration versus pore engagement for multitarget ion channel modulation remains lacking.

Here, we extend this strategy to design and evaluate new ARumenamide derivatives as multitarget modulators of Nav, Cav, and Kv channels. We tested the central hypothesis that ARumenamide derivatives act predominantly as broadly acting multitarget ion-channel modulators, rather than as strictly fenestration-selective or single-channel ligands, and that the balance between fenestration and central-pore engagement is governed by specific chemical substituents within the series. The integrated workflow described below was designed to evaluate this hypothesis by quantifying cross-family binding and by comparing fenestration versus central-pore engagement for each prioritized compound. Using structure-based docking, pharmacokinetic (ADMET) prediction, and extensive 250 ns molecular dynamics (MD) simulations, we characterized the binding of top AR candidates (AR-310, AR-769, AR-946) at both fenestration and pore sites of Nav1.4, Cav1.2, and Kv4.3. This integrated computational workflow, which combines molecular docking with dynamic simulations to refine binding hypotheses, provides mechanistic insight into ligand–channel interactions and identifies promising leads for experimental follow-up. Our findings reveal how fenestration-targeted ARs engage conserved hydrophobic pockets across channel families and suggest design principles for future multitarget ion-channel drugs.

## 2. Results

### 2.1. Molecular Docking Results

**Figure 1:**
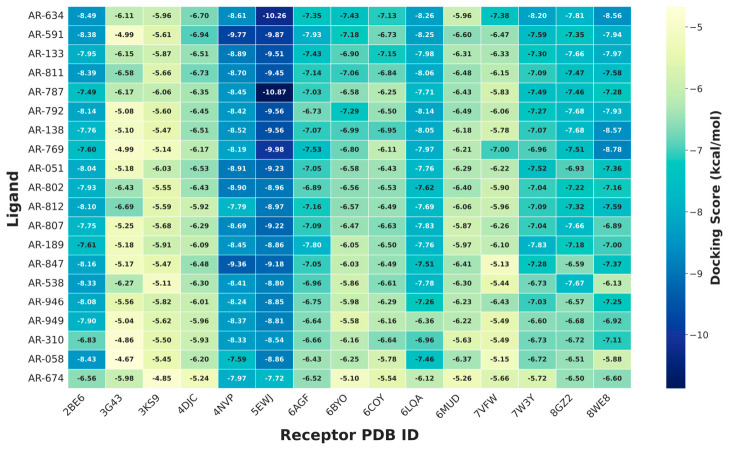
Heatmap of AutoDock Vina-predicted binding affinities (kcal/mol) for 20 ARumenamide ligands docked into fenestration sites of 15 ion channel structures. More negative values (blue) indicate stronger predicted binding, whereas less negative values (yellow) indicate weaker interactions.

Binding affinities (kcal/mol) predicted by AutoDock Vina are summarized for 20 ARumenamide ligands docked against 15 ion channel structures. The resulting heatmap visualizes relative binding strengths, with more negative values (blue) corresponding to stronger predicted interactions and less negative values (red) indicating weaker binding. Across all targets, the majority of AR compounds exhibited a range of moderate to more favorable predicted docking scores, ranging from approximately −6.0 to −10.8 kcal/mol (Figure 1). It should be emphasized that AutoDock Vina scores are empirical scoring-function estimates intended for the relative ranking of poses and compounds; they are therefore used here as a prioritization metric and are not interpreted as quantitative experimental binding affinities.

Several ligands, including AR-634, AR-591, AR-811, AR-787, AR-792, AR-538, and AR-946, consistently displayed more favorable predicted docking scores across multiple channel isoforms, with values below −8.0 kcal/mol for several targets, indicative of potential multitarget engagement. In contrast, AR-674, AR-058, and AR-310 showed weaker and more variable binding profiles, with docking scores frequently clustered between −5.0 and −6.0 kcal/mol (Figure 1).

The overall score distribution indicates that fenestration-focused docking enabled the identification of compounds with favorable interactions across both cardiac and neuronal ion channels, while preserving selectivity differences among skeletal muscle and regulatory targets. The recurrent consistently favorable docking profiles observed for AR-634, AR-591, and AR-811 suggest that shared structural features, such as aromatic moieties and substitution patterns, may contribute to broader channel compatibility. AR-634, AR-591, and AR-811 produced the most consistently favorable predicted docking scores in the screen. Nevertheless, the subsequent molecular dynamics analysis was deliberately performed on AR-310, AR-769, and AR-946. This set was chosen to represent the three target channel families and to span the spectrum of docking behaviors observed, rather than to reflect the docking-score ranking alone (see Section 4.5). The in silico ADMET evaluation, by contrast, was applied to the full 20-compound series (Figure 1).

**Figure 2:**
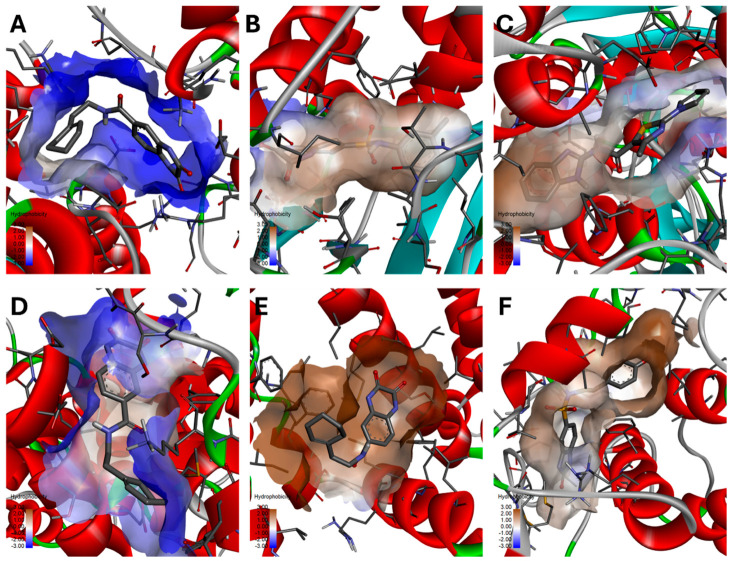
Binding modes of representative ARumenamide ligands within ion channel fenestrations. Docking poses of AR ligands in fenestration sites of sodium channel isoforms: (**A**) AR-634–Nav1.5 (2BE6), (**B**) AR-591–NavAb (4NVP), (**C**) AR-787–Nav1.4 (5EWJ), (**D**) AR-634–Nav1.5 variant (6LQA), (**E**) AR-634–Nav1.7 (7W3Y), and (**F**) AR-769–Nav1.6 (8WE8). Ligands are shown as sticks within color-coded surfaces representing local hydrophobicity.

Inspection of representative docking poses revealed distinct yet coherent binding modes across the ARumenamide series (Figure 2). AR-634 consistently adopted a well-defined orientation across multiple channel isoforms (PDB: 2BE6, 6LQA, and 7W3Y), inserting deeply into hydrophobic fenestration pockets, indicative of strong cross-target compatibility. In contrast, AR-591 (PDB: 4NVP) occupied a more solvent-exposed region and was stabilized predominantly by hydrophobic contacts, consistent with its comparatively moderate docking affinity. AR-787 (PDB: 5EWJ) formed extended interactions along the fenestration pathway, in agreement with its strong docking score and high predicted membrane permeability. AR-769 (PDB: 8WE8) localized within a cavity comprising both hydrophobic and polar residues, reflecting an intermediate binding profile (Figure 2).

These observations indicate that effective hydrophobic complementarity between AR ligands and fenestration-lining residues is a key determinant of stable binding. Among the compounds examined, AR-634 emerges as a particularly versatile multitarget binder, consistent with its favorable docking consensus scores. These structural trends provide insight into the molecular features governing ARumenamide interactions across cardiac, skeletal, and neuronal ion channel isoforms (Figure 1 and Figure 2).

**Figure 3:**
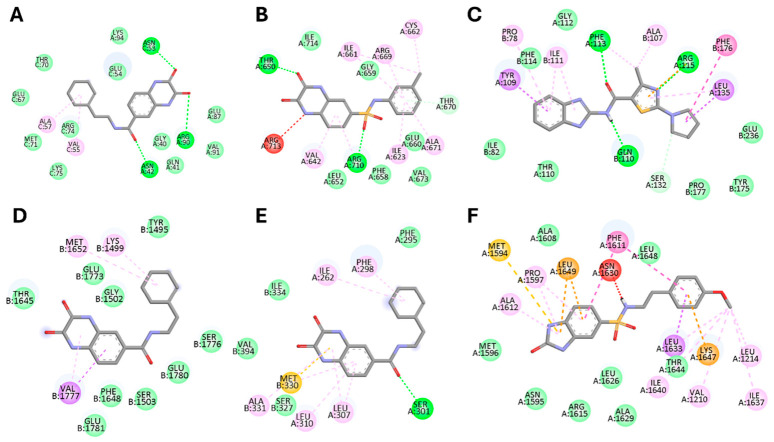
Representative docking poses of ARumenamide ligands in ion channel fenestration sites. (**A**) AR-634 bound to Nav1.5 (2BE6), showing deep insertion into a hydrophobic pocket; (**B**) AR-591 bound to NavAb (4NVP), showing a more solvent-exposed pose with predominantly hydrophobic contacts; (**C**) AR-787 bound to Nav1.4 (5EWJ), showing an extended orientation along the fenestration pathway; (**D**) AR-634 bound to a Nav1.5 variant (6LQA), showing a binding mode similar to that in panel (**A**); (**E**) AR-634 bound to Nav1.7 (7W3Y), showing compatibility across sodium channel isoforms; and (**F**) AR-769 bound to Nav1.6 (8WE8), showing binding within a mixed hydrophobic/polar cavity. Ligands are shown as sticks within color-coded surfaces representing local hydrophobicity.

The interaction maps shown in Figure 3 reveal a recurring combination of hydrophobic contacts and hydrogen bonding across the ARumenamide series. AR-634 (Figure 3A,D,E) formed multiple hydrogen bonds with polar residues (GLU, ASN, and SER) in conjunction with extensive hydrophobic interactions involving VAL and LEU side chains, consistent with its stable binding across multiple channel isoforms. AR-591 (Figure 3B) interacted with polar residues such as ARG and GLU while also engaging in aromatic stacking with PHE, indicative of a mixed hydrophilic–hydrophobic anchoring mode. AR-787 (Figure 3C) exhibited dense interaction networks with PHE, LEU, and GLN residues within the fenestration pocket, in line with its strong predicted docking affinity. In contrast, AR-769 (Figure 3F) displayed a more balanced interaction profile, combining hydrophobic contacts (ALA, LEU) with polar stabilizations (ASN, ARG), consistent with a moderate yet stable binding mode.

**Figure 4:**
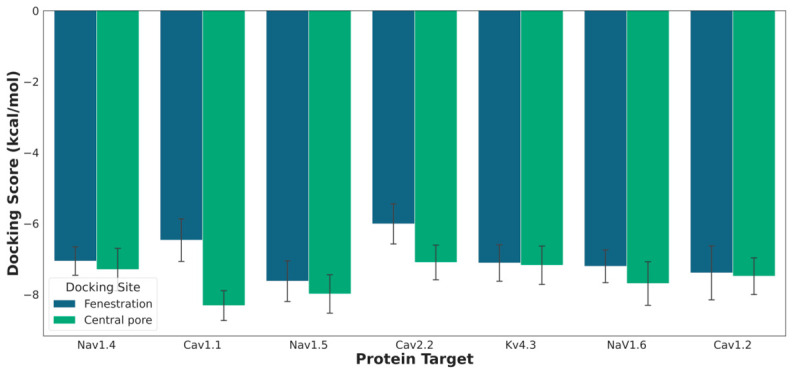
Comparison of average docking affinities for each ion channel between fenestration and central pore sites. Bars represent mean binding energies (kcal/mol) across all ligands, with lower values indicating stronger binding. Overall, central pore sites generally exhibit slightly stronger affinities than fenestration sites.

Comparative analysis of docking affinities showed that, overall, ligands tended to exhibit stronger predicted binding at central pore sites than at fenestration regions across the ion channels examined. This trend was most pronounced for calcium channels, particularly Cav1.1, Cav2.2, and Cav1.2, suggesting that their central cavities provide a more accommodating environment for ligand stabilization. In contrast, Nav1.x channels (Nav1.4, Nav1.5, and Nav1.6) displayed more modest differences between pore and fenestration binding, while Kv4.3 showed minimal variation between the two sites. Collectively, these results indicate that the central pore generally represents a more energetically favorable binding environment, consistent with its role as a structurally permissive region for ligand accommodation (Figure 4).

### 2.2. ADMET Results

**Figure 5:**
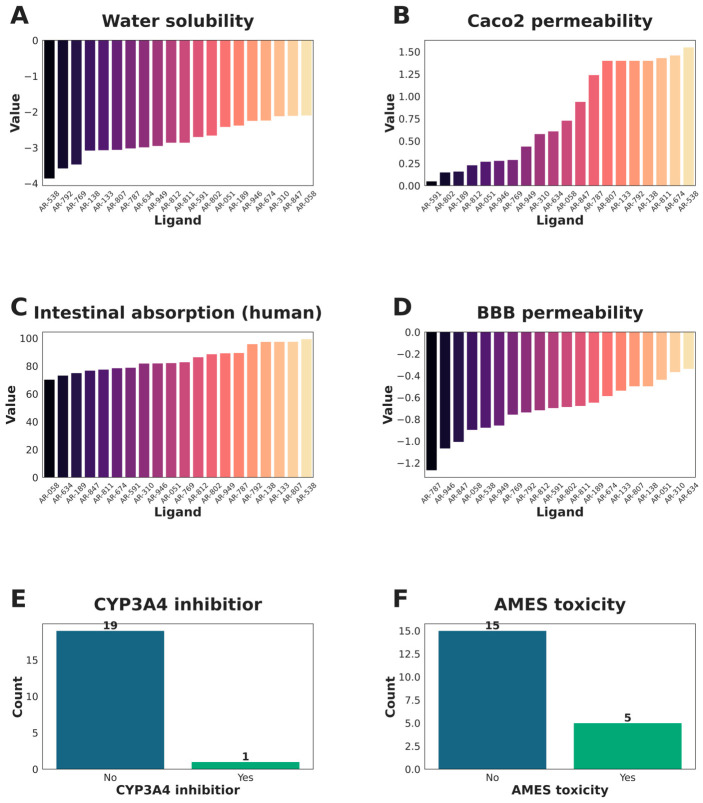
In silico-predicted ADMET properties of ARumenamide ligands. (**A**) Predicted aqueous solubility; (**B**) intestinal absorption; (**C**) blood–brain barrier permeability; (**D**) cytochrome P450 inhibition; (**E**) total clearance; and (**F**) toxicity-related endpoints. These predictions were used to prioritize compounds with favorable drug-like properties.

Computational pharmacokinetic and toxicity predictions highlight distinct trends across the ARumenamide series. Predicted aqueous solubility values were generally low (−2.5 to −4.0 log mol/L), consistent with limited dissolution potential. In contrast, Caco-2 permeability and human intestinal absorption were favorable for the majority of compounds, with several ligands (AR-133, AR-811, AR-787) achieving high predicted permeability (>1.0 log units) and absorption rates approaching 100%, suggesting robust oral bioavailability. Predictions for blood–brain barrier penetration were largely negative, indicating that most ligands are unlikely to accumulate in the central nervous system, an advantageous feature for cardiac- and peripherally directed therapeutics. The CYP3A4 inhibition profile was largely benign, with only a single ligand (AR-787) predicted to interfere with this key metabolic enzyme, thereby minimizing the likelihood of clinically significant drug–drug interactions across the series. However, Ames mutagenicity alerts were observed for several candidates (AR-138, AR-802, AR-812, AR-946), raising concerns about potential genotoxicity that warrant further experimental validation. Taken together, these ADMET predictions suggest that ARumenamides exhibit a favorable balance between predicted absorption efficiency and predicted metabolic stability, though challenges remain with respect to solubility optimization and toxicity liabilities in a subset of compounds. These insights provide a rational framework for prioritizing lead candidates for downstream medicinal chemistry refinement (Figure 5).

For interpretation, the following pkCSM reference ranges were applied. Predicted aqueous solubility (log S, mol/L) above approximately −4 indicates adequate solubility, with progressively lower values reflecting poor dissolution. Caco-2 permeability is considered high above 0.90 (log Papp, in units of 10^−6^ cm/s). Predicted human intestinal absorption below 30% is regarded as poor absorption. A blood–brain barrier value (log BB) above 0.3 indicates ready central nervous system penetration, whereas a value below −1 indicates poor penetration. Cytochrome P450 inhibition, Ames mutagenicity, and hepatotoxicity are reported as categorical (positive or negative) classifications. These endpoints provide only a comparative, computational prioritization of drug-likeness; they are model predictions rather than experimental measurements and should not be interpreted as evidence of clinical safety or efficacy, which will require dedicated in vitro and in vivo evaluation.

### 2.3. Molecular Dynamics Trajectory Analysis

#### 2.3.1. Root-Mean Square Deviation and Hydrogen Contacts

**Figure 6:**
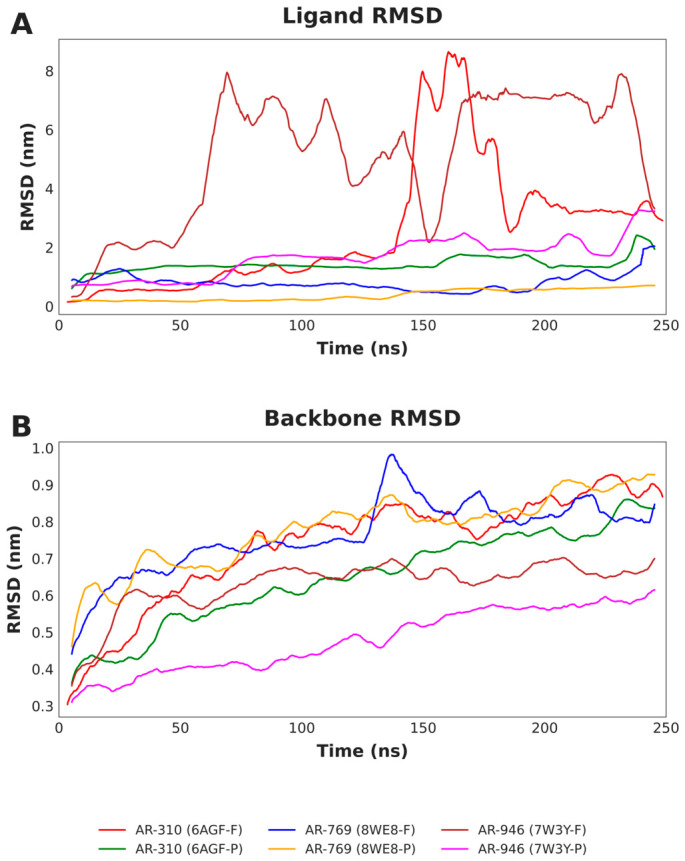
RMSD analysis of ligand binding and protein stability over 250 ns. (**A**) Ligand RMSD plots for AR-310, AR-769, and AR-946 at fenestration and central pore binding sites. High deviations (purple line) indicate ligand instability or unbinding events. (**B**) Backbone RMSD plots for the corresponding ion channels (Nav1.4, Cav1.2, Kv4.3), showing the structural deviation of the protein receptors over the simulation time.

The molecular dynamics simulations assessed structural stability using root-mean-square deviation (RMSD) analysis over the 250 ns trajectories. Backbone RMSD traces indicate that the protein–ligand complexes generally reached equilibrium, fluctuating between 0.4 and 0.9 nm. Within these single trajectories, the AR-946–Kv4.3 central-pore complex showed greater backbone rigidity than the AR-769–Cav1.2 fenestration system, which displayed transient conformational fluctuations around 130 ns. Ligand RMSD analysis indicated that AR-769 maintained stable binding (<1.0 nm deviation) at both Cav1.2 sites. In contrast, AR-310 and AR-946 behaved in a site-dependent manner: each remained stable in one environment (the Nav1.4 fenestration and the Kv4.3 central pore, respectively) but showed large RMSD excursions (>6.0 nm) in its alternative pocket. Because each system was represented by a single trajectory, these excursions are described as trajectory-specific trends consistent with reduced binding stability rather than as definitive dissociation events, and replicate simulations would be required to confirm them (Figure 6; see Section 3).

**Figure 7:**
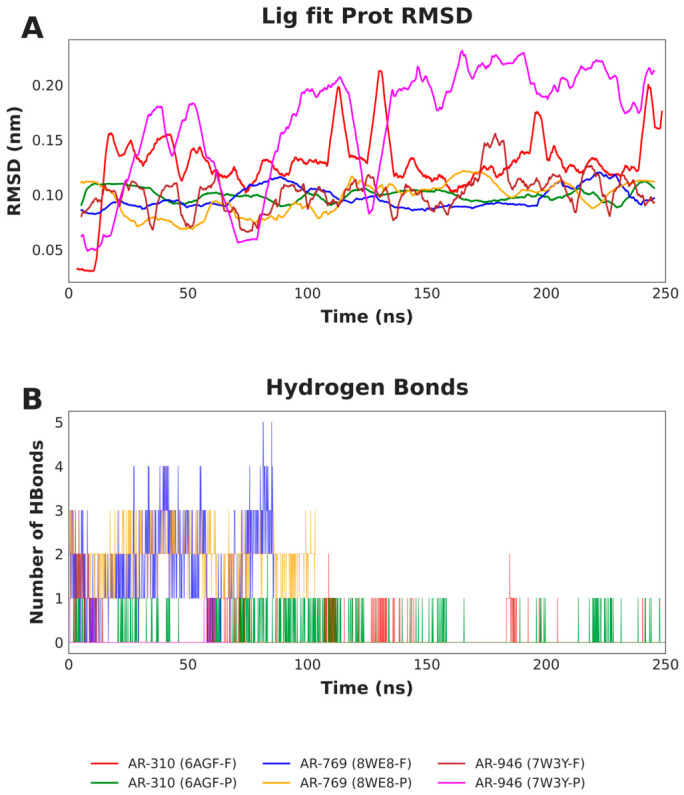
Ligand stability and interaction dynamics. (**A**) Ligand RMSD relative to the protein backbone; low, flat traces (e.g., AR-769) indicate stable binding, while large spikes (e.g., AR-946) reflect dissociation. (**B**) Hydrogen bond evolution, showing that sustained H-bond networks (e.g., green/orange traces) underpin the structural stability observed in panel (**A**).

To evaluate the dynamic stability and interaction durability of the top-ranked complexes, we analyzed the Ligand-fit-to-Protein RMSD and the time evolution of hydrogen bonds over the 250 ns trajectory (Figure 7A,B). The analysis reveals a distinct correlation between structural stability and the persistence of intermolecular hydrogen bonds.

AR-769 exhibited the most robust binding profile within Cav1.2, particularly at the fenestration site (green trace), where it maintained a low and stable RMSD (<1.0 nm) anchored by a dense network of 2–5 persistent hydrogen bonds during the first 100 ns. Similarly, the central pore complex (orange trace) remained highly stable (RMSD ~0.2–0.5 nm) with a steady maintenance of 1–3 hydrogen bonds, confirming high-affinity retention (Figure 7).

In contrast, AR-310 and AR-946 displayed site-dependent instability. While AR-310 remained relatively stable in the Nav1.4 fenestration (RMSD ~1.5 nm), the central pore-bound ligand (blue trace) became unstable after approximately 140 ns, as indicated by a sharp RMSD increase (>8.0 nm) and a loss of hydrogen-bond contacts. A comparable loss of stable occupancy was observed for AR-946 in the Kv4.3 fenestration (purple trace), where early fluctuations (>6.0 nm) and few hydrogen bonds indicated a failure to establish stable binding within this single trajectory. These results suggest that the sustained engagement of multiple hydrogen bonds is a critical determinant for preventing ligand egress and maintaining stable binding in these ion channel targets (Figure 7). That these large RMSD excursions reflect genuine loss of the native binding pose, rather than internal reorientation of the ligand within an intact pocket, is supported by their close temporal coincidence with the collapse of the protein–ligand hydrogen-bond network shown in Figure 7B. For both AR-310 (Nav1.4 central pore) and AR-946 (Kv4.3 fenestration), the sharp rise in ligand RMSD and the loss of hydrogen-bond contacts occur together within the same window of the trajectory, indicating that the ligand departed from its initial binding site. The convergence of these two independent metrics, ligand RMSD and hydrogen-bond persistence, provides internal corroboration of the dissociation trend, which the planned replicate simulations are intended to confirm.

#### 2.3.2. Root-Mean Square Fluctuations

To assess how ligand binding affects local protein flexibility, root mean square fluctuation (RMSF) profiles were calculated on a per-residue basis. The resulting patterns reveal distinct dynamic behaviors associated with binding site occupancy.

For Nav1.4, the AR-310–fenestration complex (red trace) showed consistently lower residue fluctuations across the sequence compared with the central pore complex (blue trace). Reduced flexibility was particularly evident in loop regions spanning residues 450–550 and 1550–1600, consistent with a more rigid protein framework and in agreement with the stable ligand RMSD observed previously. In contrast, central pore binding was associated with increased protein mobility, which likely contributes to the ligand dissociation event detected in that system.

In the Cav1.2–AR-769 complexes, RMSF profiles were largely comparable between fenestration and central pore binding modes, reflecting the overall stability of this ligand–channel interaction. Nevertheless, fenestration binding (green trace) was associated with increased flexibility at the C-terminal region around residue 1600, suggesting a localized allosteric response distinct from the relatively rigid transmembrane core.

**Figure 8:**
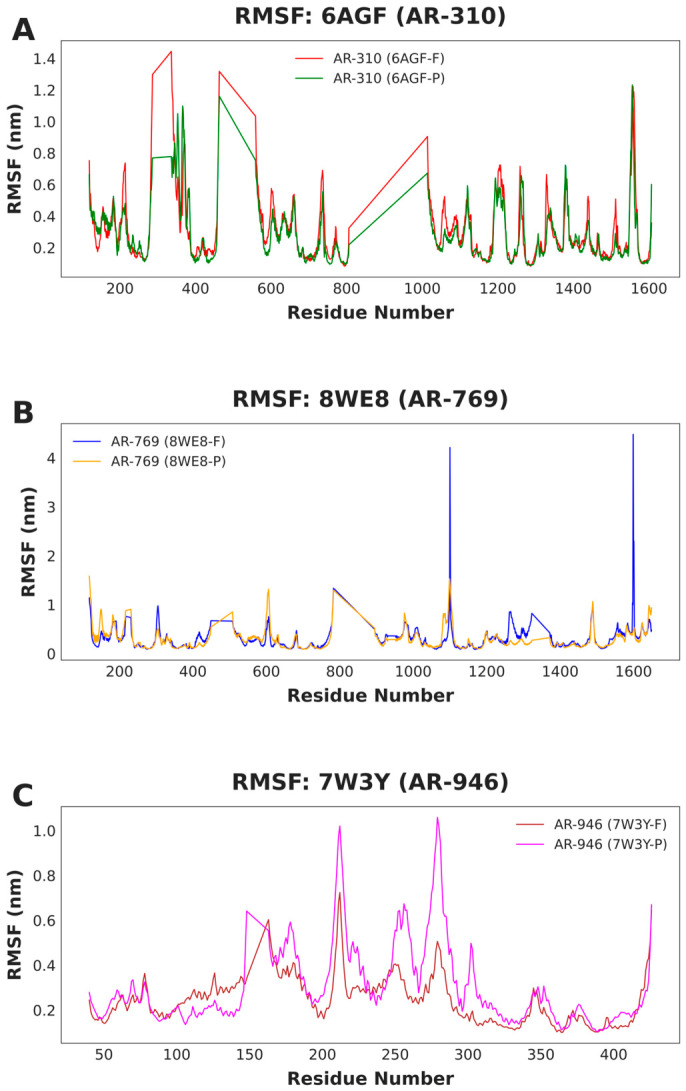
Root Mean Square Fluctuation (RMSF) analysis of protein residues. The plots illustrate the local flexibility of the protein backbone for (**A**) Nav1.4, (**B**) Cav1.2, and (**C**) Kv4.3 upon ligand binding. High RMSF values (peaks) correspond to flexible regions such as loops and termini, while low values (valleys) indicate rigid transmembrane domains.

In Kv4.3, stable binding of AR-946 within the central pore (brown trace) resulted in higher overall residue fluctuations than those observed for the fenestration-bound complex (purple trace). This behavior is consistent with an induced-fit mechanism in which pore binding accommodates the ligand through sustained local flexibility, whereas the less stable fenestration interaction fails to promote a comparable dynamic adaptation (Figure 8).

#### 2.3.3. Solvent-Accessible Surface and Radius of Gyration 

To assess global structural changes and solvent exposure induced by ligand binding, we analyzed the Radius of Gyration (Rg) and Solvent Accessible Surface Area (SASA). The trajectories indicate that while most systems maintained a steady global conformation, distinct ligand-induced effects were observed for Cav1.2.

The Cav1.2–AR-769 complex bound at the central pore (orange trace) exhibited a significant compaction event, characterized by a concurrent decrease in Rg (from ~3.64 to ~3.55 nm) and SASA (from ~690 to ~640 nm^2^) beyond 100 ns. This reduction suggests a "tightening" of the pore or an induced-fit mechanism where the channel closes around the ligand, stabilizing the complex.

**Figure 9:**
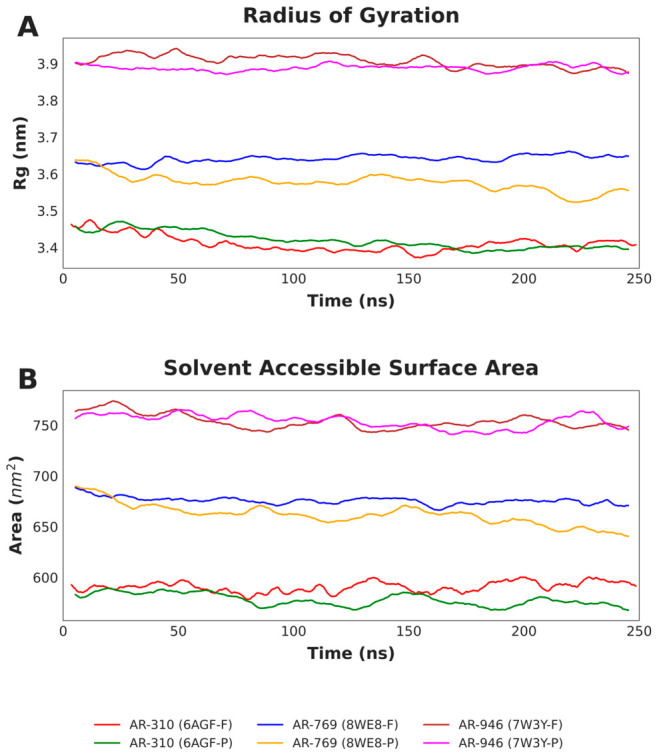
Protein compactness and surface exposure during molecular dynamics simulations. (**A**) Time evolution of the radius of gyration (Rg), reflecting global protein compactness. Decreasing Rg values indicate progressive structural compaction. (**B**) Solvent-accessible surface area (SASA) as a measure of protein exposure to solvent, with lower values corresponding to more compact or closed conformational states.

In the case of Nav1.4, binding at the fenestration (red trace) resulted in consistently lower SASA values compared to the central pore (blue trace), indicating a more compact, less solvent-exposed state that aligns with the higher stability observed in earlier RMSD metrics. Meanwhile, Kv4.3 complexes remained the most expanded (Rg ~3.9 nm) due to the channel’s large tetrameric structure. However, the central pore-bound system (brown trace) displayed remarkable stability in both Rg and SASA, whereas the fenestration-bound system (purple trace) showed greater fluctuations, reflecting a more dynamic and less structurally defined state (Figure 9).

#### 2.3.4. Free Energy Landscapes

To further elucidate the structural mechanisms underlying the stability of the protein–ligand complexes, Free Energy Landscapes (FEL) were constructed using Principal Component Analysis (PCA) of the backbone trajectories. The topology of these energy basins provides a direct visualization of the conformational entropy and stability of each system.

The Cav1.2–AR-769 complex bound at the central pore (middle right panel) exhibits a distinctive "funnel-like" energy landscape, characterized by a single, deep, and narrow basin. This topology indicates that the protein is confined to a highly restricted conformational subspace, consistent with the “tightening” observed in the Radius of Gyration analysis and the high affinity interactions discussed previously.

**Figure 10:**
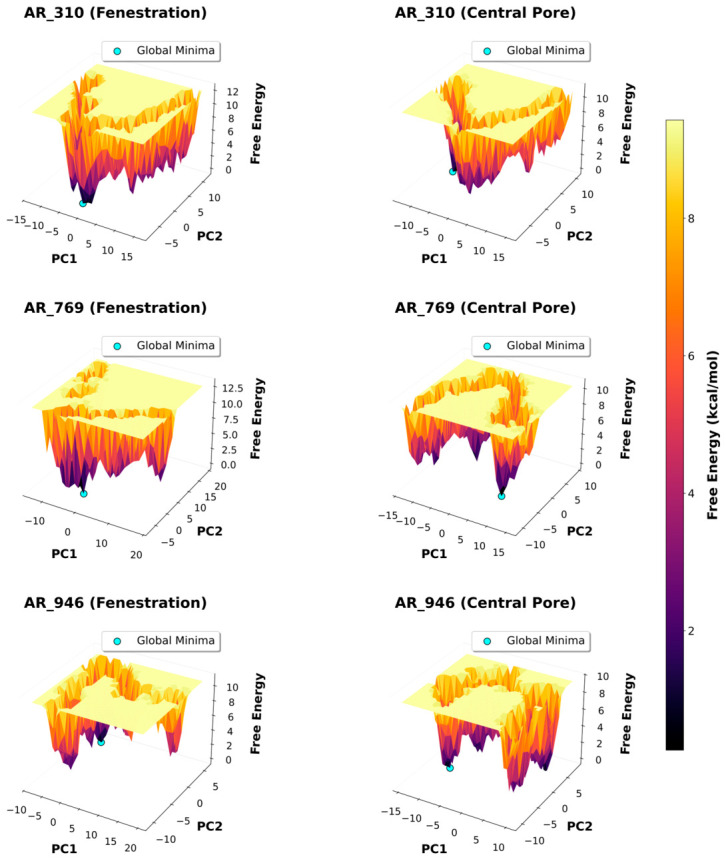
Free Energy Landscapes (FEL) of protein conformational dynamics. The 3D surface plots represent the conformational space sampled by the protein backbones during the 250 ns simulation, projected onto the first two principal components (PC1 vs. PC2). The color gradient indicates the relative Gibbs free energy, where dark blue basins represent stable, low-energy conformational states (global minima marked by red dots) and yellow peaks represent higher-energy transient states. Because each landscape was derived from a single 250 ns trajectory, these plots should be interpreted as qualitative maps of the conformational space sampled rather than as fully converged, quantitative free energy surfaces.

In comparison, the Nav1.4 systems display site-specific differences. The fenestration-bound complex (top left panel, Figure 10) resides in a relatively well-defined energy minimum, suggesting conformational rigidity induced by the ligand. Conversely, the central pore complex (top right panel, Figure 10) and the Kv4.3 fenestration system (bottom left panel, Figure 10) display broader and more rugged conformational basins. This structural heterogeneity implies that these proteins sample a wider range of conformations, likely due to the lack of stable ligand locking or the enhanced flexibility associated with the ligand dissociation events observed in the RMSD analysis (Figure 10). Because these landscapes were derived from single 250 ns trajectories, they should be interpreted as a qualitative representation of the conformational space sampled within each simulation rather than as fully converged free energy surfaces; replicate simulations would be required to establish their quantitative robustness (see Section 3).

## 3. Discussion

Our molecular dynamics simulations provide a mechanistic extension of the docking and ADMET analyses, helping to rationalize why certain ARumenamide derivatives display broader multi-channel engagement whereas others exhibit more selective behavior. In particular, AR-769 showed sustained and stable binding at both fenestration and central pore sites of Cav1.2, with the fenestration-bound state being especially persistent. This stability was supported by a dense network of hydrophobic interactions and hydrogen bonds and was accompanied by reduced radius of gyration and a well-defined free energy minimum. Such ligand-induced conformational tightening has been reported previously for small-molecule modulators of voltage-gated ion channels and is consistent with induced-fit mechanisms described for sodium and calcium channel blockers [15,17].

In contrast, AR-310 and AR-946 displayed stability only within specific binding environments. AR-310 was preferentially retained at the Nav1.4 fenestration while showing instability in the central pore. This site preference suggests that subtle chemical modifications can bias a ligand toward lateral entry pathways rather than pore-facing sites. The trend is in line with prior structure–activity relationships reported for ARumenamides, in which aromatic substitution patterns influenced fenestration residency and reduced pore block [16]. AR-946, by contrast, showed selective stability within the Kv4.3 central pore but not at the fenestration. This distinction is particularly relevant given the established role of Kv4.3-mediated Ito in shaping early cardiac repolarization and arrhythmogenic substrates [18].

Loss of binding stability at non-preferred sites for AR-310 and AR-946 correlated with reduced hydrogen bond persistence and increasing ligand RMSD, indicating that insufficient polar anchoring may facilitate ligand egress. Similar relationships between hydrogen bond lifetimes and ligand retention have been observed in molecular dynamics studies of sodium channel blockers and other membrane-embedded targets, reinforcing the importance of balanced hydrophobic and polar interactions for sustained binding [19].

Comparison with experimental literature further supports the relevance of these findings. AR-787, a closely related ARumenamide analog, has been shown experimentally to modulate multiple cardiac ion channels, enhancing Nav1.5 current while inhibiting Ito, IKr, and ICa in vitro [16]. In this context, our simulations suggest that AR-769 may possess analogous multitarget potential, particularly through Cav1.2 engagement, whereas AR-310 and AR-946 appear more selective for Nav1.4 and Kv4.3, respectively. The binding modes observed here—fenestration versus pore occupancy, induced-fit behavior, and reliance on hydrogen bond networks—are consistent with established drug-binding paradigms for voltage-gated ion channels, including lateral access pathways originally proposed by Hille and subsequently confirmed by structural and functional studies [9,20].

From a translational perspective, the predicted ADMET profiles of the ARumenamides, characterized by favorable predicted membrane permeability and generally low predicted toxicity, are consistent with drug-like physicochemical behavior and provide a preliminary, computational basis for prioritizing these compounds for further study. Importantly, the simulations suggest that strong fenestration binding can reduce overall channel flexibility, as reflected by lower RMSF values, which may help stabilize gating behavior without complete pore occlusion. Conversely, pore binding that preserves a degree of conformational flexibility, as observed for Kv4.3, may be associated with less pronounced channel block. Because the present study is entirely computational and does not include electrophysiological or toxicity measurements, these translational implications remain hypotheses that require direct experimental validation.

Several limitations of the present computational workflow should be acknowledged. First, the molecular dynamics simulations were performed with the protein–ligand complexes solvated in explicit water rather than embedded in an explicit lipid bilayer. Because voltage-gated ion channels are integral membrane proteins whose transmembrane helices and lateral fenestrations are normally stabilized by, and accessed through, the surrounding lipid phase, a water-only environment may overestimate the flexibility of hydrophobic transmembrane regions and does not reproduce lipid-mediated access pathways to the fenestrations. The binding behaviors reported here should therefore be regarded as a first-pass assessment of intrinsic site complementarity rather than a complete description of membrane-embedded dynamics, and future work will repeat the prioritized simulations in explicit bilayers constructed with tools such as CHARMM-GUI Membrane Builder [21]. Second, the staged equilibration protocol (100 ps NVT followed by 100 ps NPT) is relatively short for large, flexible channel proteins; although the extended 250 ns production runs permit substantial further relaxation, longer multi-stage equilibration with gradual restraint release would provide additional confidence in the starting ensembles. Third, each system was simulated as a single 250 ns trajectory without independent replicas or enhanced sampling. As a consequence, the free energy landscapes presented in Section 2.3.4 should be interpreted as qualitative descriptions of the conformational space sampled within each run rather than as fully converged surfaces, and replicate simulations will be needed to establish the statistical robustness of the observed stability and dissociation events [22]. Fourth, the ion channel structures used for docking span a range of experimental resolutions, and the lower-resolution cryo-EM models in particular carry greater uncertainty in side-chain placement; the predicted hydrogen-bond networks and binding energies derived from these structures should accordingly be treated as hypotheses for experimental testing, and a systematic sensitivity analysis across alternative structures and conformational states represents an important direction for future refinement. Despite these limitations, the convergent trends observed across the docking, ADMET, and molecular dynamics analyses provide a coherent and experimentally testable framework for prioritizing ARumenamide derivatives for functional validation.

In summary, this study integrates structure-based docking, in silico ADMET profiling, and molecular dynamics simulations to computationally prioritize ARumenamide derivatives as candidate multitarget ion channel modulators. The mechanistic insights gained into binding stability, conformational effects, and interaction fingerprints provide a rational, hypothesis-generating foundation for the further design and optimization of AR compounds. Future work should focus on electrophysiological validation of the prioritized candidates to test their predicted effects on Nav1.4, Cav1.2, and Kv4.3 currents and to evaluate their possible relevance to cardiac and neuromuscular disorders. Collectively, these computational results highlight the value of combining biophysical simulations with pharmacological context when prioritizing polypharmacological ion channel modulators for experimental study.

## 4. Materials and Methods

### 4.1. Protein Preparation

Fifteen ion channel targets relevant to cardiac, neuronal, and skeletal muscle physiology, as well as cross-tissue regulatory mechanisms, were retrieved from the RCSB Protein Data Bank (PDB) [23]; Table 1. Structures were selected based on the availability of resolved transmembrane domains, the presence of resolved or inferred fenestration pathways, and their relevance to clinically validated channelopathies. Preference was given to open or inactivated conformational states, which are known to expose lateral fenestrations and thus facilitate access by lipophilic ligands. The selected structures spanned a range of experimental resolutions; the potential influence of structural resolution on docking accuracy and on the inferred protein–ligand interaction networks is addressed in the Discussion (Section 3). Although the principal targets of this study are voltage-gated sodium, calcium, and potassium channels, the structure set also deliberately includes ionotropic and metabotropic glutamate receptors and four Ca^2+^–calmodulin-bound regulatory domains. These additional structures were retained as mechanistic comparators rather than as primary targets. The glutamate receptors are non-voltage-gated channels that allow the fenestration-targeting behaviour of ARumenamides to be tested for selectivity against an unrelated channel architecture. The Ca^2+^–calmodulin-bound IQ-domain and C-terminal structures are physiologically important cross-tissue regulatory modules that interface directly with the cardiac sodium and calcium channels examined here. Their inclusion therefore probes whether ARumenamide binding is specific to voltage-gated pore-forming domains or also extends to associated regulatory and non-voltage-gated proteins, and the corresponding docking and dynamics results are interpreted in that comparative context (Section 2 and Section 3).

Prior to molecular docking, all protein structures were subjected to standardized preprocessing and optimization. Missing loop regions were modeled using MODELLER v10.7 [24], and protonation states of titratable residues were assigned using the H++ server (http://newbiophysics.cs.vt.edu/H++/, accessed on 17 January 2026) at physiological pH [25]. Non-standard residues and crystallographic water molecules were removed. Atom types and Gasteiger partial charges were then assigned using ForliLab’s Meeko package, and the prepared structures were converted to PDBQT format for subsequent docking simulations [26].

### 4.2. Ligand Dataset Preparation 

Twenty ARumenamide (AR) compounds were selected from the original discovery and characterization study by Abdelsayed et al. (2022) based on their reported chemical identities and fenestration-targeting potential [16]. The compounds were defined using their published IUPAC names and converted into canonical SMILES strings using the Open Parser for Systematic IUPAC Nomenclature (OPSIN). The resulting SMILES were standardized and converted into MOL2 format using OpenBabel [27], followed by three-dimensional structure generation and geometry optimization employing the MMFF94 force field. The optimized ligand structures were subsequently converted to PDBQT format using Meeko package [26], during which atom types were assigned and Gasteiger partial charges were calculated for compatibility with AutoDock-based molecular docking simulations [28]. Protonation states were assigned at physiological pH (≈7.4) during ligand preparation; where more than one tautomer was chemically plausible, the lowest-energy tautomer obtained after MMFF94 geometry optimization was retained; and all stereocentres were fixed in accordance with the stereodescriptors specified in the published IUPAC names reported by Abdelsayed et al. (2022) [16]. The resulting net formal charges followed directly from the assigned protonation states.

### 4.3. Molecular Docking

The selected ion channel structures were analyzed for binding site accessibility using CAVER Web, with tunnel identification and prioritization based on geometric properties and predicted druggability scores. Fenestration docking grids were centered on CAVER-identified lateral tunnels exhibiting high druggability, ensuring unbiased and structure-guided targeting of fenestration sites. Central pore docking was performed exclusively as a comparative reference to evaluate relative binding energetics and was not the primary focus of the screening strategy. Accordingly, fenestration regions were prioritized as the principal docking targets throughout this study. For every structure, fenestration sites were defined using the same selection criterion: among the lateral tunnels identified by CAVER Web that connected the lipid-facing surface to the central cavity, the tunnel (or tunnels) with the highest predicted druggability score was retained, and the docking grid box was centred on the bottleneck region of the selected tunnel. This identical criterion was applied consistently across all fifteen structures to avoid target-specific bias in fenestration-site definition.

Docking grid box centre coordinates (x, y, z) and dimensions for both the fenestration and central-pore docking sites of all fifteen channel structures, together with the CAVER tunnel identifier and predicted druggability score, are used to define each fenestration site and the residues delimiting each central-pore site. Molecular docking was carried out using AutoDock Vina v1.2.0 within a Conda-managed environment, employing an exhaustiveness value of 16 and generating up to 10 binding poses per ligand. Binding affinities for all protein–ligand complexes were systematically collected and compiled into comma-separated value (CSV) files for downstream analysis. Docking poses and protein–ligand interaction patterns were subsequently examined using Discovery Studio Visualizer.

### 4.4. ADMET Prediction

To evaluate the pharmacological plausibility of the candidate ligands, their absorption, distribution, metabolism, excretion, and toxicity (ADMET) profiles were systematically assessed in silico. These properties are primarily determined by intrinsic physicochemical descriptors and molecular fingerprints, which influence interactions with biological membranes, transporters, and metabolic enzymes involved in drug disposition and clearance. ADMET predictions were performed using the pkCSM web server [29], which employs graph-based signatures to estimate key pharmacokinetic and toxicity parameters. The evaluated endpoints included aqueous solubility, blood–brain barrier permeability, cytochrome P450 enzyme inhibition, total systemic clearance, as well as predicted Ames mutagenicity and hepatotoxicity. This in silico screening provided a comparative framework for prioritizing compounds with favorable drug-like characteristics while acknowledging the inherent limitations of predictive ADMET models.

### 4.5. Molecular Dynamics Simulations

Three ARumenamide derivatives—AR-310, AR-769, and AR-946—were prioritized for 250 ns molecular dynamics (MD) simulations to characterize the stability and dynamics of their interactions with ion channel targets. Rather than restricting the dynamic analysis to the highest-scoring docking ligands, these three compounds were selected to represent each of the three target channel families examined here (Nav1.4, Cav1.2, and Kv4.3) and to span the range of docking behaviors observed across the series—from a consistent, high-affinity multitarget binder (AR-769) to ligands with more variable or moderate docking profiles (AR-310 and AR-946)—thereby enabling a direct test of whether docking-predicted affinity translates into sustained dynamic stability at fenestration versus central pore sites. Protein–ligand complexes corresponding to both fenestration and central pore binding modes were prepared using the CHARMM-GUI web server with the CHARMM36m force field [30,31]. During system setup, the “Generate PBC FFT” option was enabled to automatically define FFT grid dimensions (Nx, Ny, Nz) compatible with the Particle Mesh Ewald (PME) method, ensuring consistency with periodic boundary conditions and an approximate grid spacing of 1.0 Å.

Each system was solvated in a rectangular box of TIP3P water with a minimum solute–box distance of 10 Å. Sodium and chloride ions were added using a Monte Carlo placement scheme to neutralize the system and achieve a physiological ionic strength of 0.15 M while minimizing steric clashes. Energy minimization was performed using the steepest descent algorithm until the maximum force fell below 1000 kJ·mol^−1^·nm^−1^. Equilibration was then carried out in two stages: a 100 ps NVT simulation at 300 K using the velocity-rescaling (V-rescale) thermostat, followed by a 100 ps NPT simulation at 1 bar using the Parrinello–Rahman barostat. Following equilibration, the stability of each system was verified by inspection of the backbone RMSD time series prior to production analysis; the length of the staged-equilibration protocol and the use of single production trajectories are considered among the methodological limitations discussed in Section 3. Production MD simulations were subsequently performed for 250 ns using OpenMM [32]. Trajectory files were converted to GROMACS-compatible formats for post-simulation analyses [33]. Force-field parameters for the ARumenamide ligands were generated with the CHARMM General Force Field (CGenFF) through the CHARMM-GUI interface, ensuring consistency with the CHARMM36m parameters used for the protein. Production dynamics were propagated with a 2 fs integration timestep, with all bonds involving hydrogen atoms constrained and water molecules held rigid. Short-range van der Waals and electrostatic interactions were truncated at 1.2 nm, with a force-based switching function applied from 1.0 nm, while long-range electrostatics were evaluated using the Particle Mesh Ewald method with the FFT grid and approximate 1.0 Å spacing defined above. Production simulations were conducted in the NPT ensemble at 300 K and 1 bar, and atomic coordinates were saved every 100 ps for subsequent analysis.

All post-simulation analyses were carried out on the GROMACS-formatted trajectories using standard GROMACS utilities. Prior to analysis, periodic boundary artefacts were removed and each trajectory was least-squares fitted to the energy-minimized starting structure on the protein backbone atoms (N, Cα, C). Protein RMSD was computed for backbone atoms; ligand RMSD was computed for ligand heavy atoms after fitting on the protein backbone; and per-residue RMSF was calculated for Cα atoms over the equilibrated portion of each trajectory. Hydrogen bonds were enumerated using the default geometric criteria of a donor–acceptor distance ≤ 0.35 nm and a hydrogen–donor–acceptor angle ≤ 30°. The radius of gyration was calculated over all protein atoms, and the solvent-accessible surface area was calculated for the protein using a 0.14 nm solvent-probe radius. Principal component analysis was performed by constructing and diagonalizing the covariance matrix of the protein backbone Cartesian coordinates, and free energy landscapes were derived from the bivariate probability distribution of the first two principal components (PC1, PC2) using the relation ΔG = −kBT ln[P(PC1,PC2)/Pmax], with the probability P estimated from a two-dimensional histogram of the trajectory projected onto PC1 and PC2. Because each landscape is based on a single trajectory, it is presented as a qualitative map of the conformational space sampled rather than as a fully converged free energy surface.

## 5. Conclusions

In this study, we combined structure-based docking, in silico ADMET profiling, and long-timescale molecular dynamics simulations to investigate ARumenamide derivatives as multitarget modulators of voltage-gated ion channels. By explicitly comparing fenestration and central pore binding modes across sodium, calcium, and potassium channel isoforms, we provide mechanistic insight into how binding site selection, interaction networks, and channel conformational responses govern ligand stability and selectivity. Our results demonstrate that fenestration-targeted binding can support sustained ligand engagement and channel rigidification without obligatory pore occlusion, whereas central pore binding may preserve conformational flexibility depending on channel type and ligand chemistry. Among the compounds examined, AR-769 emerged as a promising multitarget candidate with stable engagement of Cav1.2, while AR-310 and AR-946 displayed more selective behavior toward Nav1.4 and Kv4.3, respectively. These distinct profiles highlight how subtle chemical features can bias ligands toward specific access pathways and functional outcomes. Together, these findings establish a structure–dynamics framework for rationally designing ARumenamide-based ion channel modulators with tunable selectivity and polypharmacological potential. Future experimental validation using electrophysiological assays will be essential to confirm the predicted functional effects and to determine whether these compounds merit further development as modulators of cardiac and neuromuscular channelopathies.

## Figures and Tables

**Table 1:** Selected Ion Channel Isoforms by Tissue Type.

Tissue Type	Channel Class	Notable Isoforms/Examples	PDB
Cardiac	Voltage-Gated Na^+^	Nav1.5 (SCN5A), adult cardiac isoform	6LQA, resolution = 3.3 Å
	Voltage-Gated Ca^2+^	Cav1.2 (CACNA1C), L-type calcium channel	8WE8, resolution = 2.9 Å
	Voltage-Gated K^+^	Kv4.3 (KCND3), mediator of transient outward current (Ito)	7W3Y, resolution = 3.0 Å
	Pacemaker (If/Ih)	HCN4, predominant sinoatrial node isoform	4NVP, resolution = 2.5 Å
Neuronal	Voltage-Gated Na^+^	Nav1.6 (SCN8A), central nervous system sodium channel	8GZ2, resolution = 3.3 Å
	Voltage-Gated Ca^2+^	Cav2.2 (CACNA1B), N-type calcium channel	7VFW, resolution = 3.3 Å
	Neurotransmitter-gated channels	Ionotropic glutamate receptor (AMPA-type)	5EWJ, resolution = 2.7 Å
		Metabotropic glutamate receptor	3KS9, resolution = 1.9 Å
Skeletal Muscle	Voltage-Gated Na^+^	Nav1.4 (SCN4A), skeletal muscle sodium channel	6AGF, resolution = 3.2 Å
	Voltage-Gated Ca^2+^	Cav1.1, dihydropyridine receptor	6BYO, resolution = 3.6 Å
	Cl^−^ Channel	ClC-1 (CLCN1), transmembrane domain	6COY, resolution = 3.3 Å
Cross-Tissue/Regulation	Subunits & Modulators	Nav1.5 DIII–IV linker bound to Ca^2+^–calmodulin	4DJC, resolution = 1.3 Å
		IQ domain bound to Ca^2+^–calmodulin	2BE6, resolution = 2.0 Å
		C-terminal domain bound to CaM dimer	3G43, resolution = 2.1 Å
		C-terminal domain bound to CaM	6MUD, resolution = 2.7 Å

Targets are grouped by tissue context. The voltage-gated sodium (Nav), calcium (Cav), and potassium (Kv) channels, together with the HCN4 pacemaker channel and the ClC-1 chloride channel, constitute the primary ion-channel targets of this study. The ionotropic (AMPA-type) and metabotropic glutamate receptors are non-voltage-gated channels included as architectural comparators. The four Ca^2+^–calmodulin-bound structures listed under Cross-Tissue/Regulation (the Nav1.5 DIII–IV linker, the IQ domain, and the C-terminal domains) are regulatory modules included to probe cross-tissue regulatory interactions and are not independent voltage-gated channels. Docking and molecular dynamics results for these proteins and regulatory structures are interpreted accordingly in Section 2 and Section 3.

## Data Availability

The original contributions presented in this study are included in the article. Further inquiries can be directed to the corresponding author.
